# Differential expression of circulating miRNAs after alemtuzumab induction therapy in lung transplantation

**DOI:** 10.1038/s41598-022-10866-w

**Published:** 2022-04-30

**Authors:** A. Benazzo, S. Bozzini, S. Auner, H. Oya Berezhinskiy, M. L. Watzenboeck, S. Schwarz, T. Schweiger, W. Klepetko, T. Wekerle, K. Hoetzenecker, F. Meloni, P. Jaksch

**Affiliations:** 1 Department of Thoracic Surgery, Medical University of Vienna, Vienna, Austria; 2 Department of Internal Medicine, Unit of Respiratory Diseases, Laboratory of Cell Biology and Immunology, University of Pavia and IRCCS Policlinico San Matteo Foundation, Pavia, Italy; 3 Department of Biomedical Imaging and Image-Guided Therapy, Medical University of Vienna, Vienna, Austria; 4 Section of Transplantation Immunology, Division of Transplantation, Department of General Surgery, Medical University of Vienna, Vienna, Austria; 5 Department of Thoracic Surgery, Lung Transplantation Research Lab, Medical University of Vienna, Vienna, Austria; 6 Division of Thoracic Surgery, Medical University of Vienna, Währinger Guertel 18-20, 1090 Vienna, Austria

**Keywords:** Biomarkers, Medical research, Molecular medicine, Pathogenesis

## Abstract

Alemtuzumab is a monoclonal antibody targeting CD52, used as induction therapy after lung transplantation (LTx). Its engagement produces a long-lasting immunodepletion; however, the mechanisms driving cell reconstitution are poorly defined. We hypothesized that miRNAs are involved in this process. The expression of a set of miRNAs, cytokines and co-signaling molecules was measured with RT-qPCR and flow cytometry in prospectively collected serum samples of LTx recipients, after alemtuzumab or no induction therapy. Twenty-six LTx recipients who received alemtuzumab and twenty-seven matched LTx recipients without induction therapy were included in the analysis. One year after transplantation four miRNAs were differentially regulated: miR-23b (*p* = 0.05) miR-146 (*p* = 0.04), miR-155 (*p* < 0.001) and miR-486 (*p* < 0.001). Expression of 3 miRNAs changed within the alemtuzumab group: miR-146 (*p* < 0.001), miR-155 (*p* < 0.001) and miR-31 (*p* < 0.001). Levels of IL-13, IL-4, IFN-γ, BAFF, IL-5, IL-9, IL-17F, IL-17A and IL-22 were different one year after transplantation compared to baseline. In no-induction group, concentration of sCD27, sB7.2 and sPD-L1 increased overtime. Expression of miR-23b, miR-146, miR-486, miR-155 and miR-31 was different in LTx recipients who received alemtuzumab compared to recipients without induction therapy. The observed cytokine pattern suggested proliferation of specific B cell subsets in alemtuzumab group and co-stimulation of T-cells in no-induction group.

## Introduction

Current immunosuppression (IS) protocols in lung transplantation (LTx) usually consists of an induction therapy and a triple-drug maintenance immunosuppression including a calcineurin-inhibitor (CNI), an antiproliferative drug and steroids^1^. Currently, interleukin-2 (IL-2) receptor antagonists are used by the majority of centers as induction agents^1^, followed by anti-thymocyte globulin (ATG) and alemtuzumab. Alemtuzumab is a humanized monoclonal antibody targeting CD52, a surface receptor expressed on CD4^+^ and CD8^+^ lymphocytes, Natural Killer (NK) cells and monocytes. Its activation induces cell lysis leading to an immune cell depletion lasting 6–24 months^2,3^. Although composition of T and B cell populations after alemtuzumab induction in transplantation has been described in a few studies, the underlying mechanisms driving cell reconstitution are still unknown. Alemtuzumab was introduced in our clinical routine more than 10 years ago following a prospective randomized controlled trial comparing alemtuzumab and ATG^4^. This study showed a reduced incidence of higher grade acute cellular rejection (ACR) in the alemtuzumab group. Similarly, other groups observed superiority of alemtuzumab in terms of graft and patient survival^5,6^. The mechanisms underlying the effects of alemtuzumab in the early induction phase as well as in the later immune reconstitution phase have not been clarified yet and a better knowledge of the key molecular players could open up further therapeutic possibilities.

MicroRNAs (miRNAs) are evolutionarily conserved small non-coding RNAs, which function as post-transcriptional negative regulators of target mRNAs^7^. As such, they play a fundamental role as orchestrators of complex biological processes including cell differentiation and apoptosis^7^. Aberrant expression of miRNAs is associated with initiation and progression of pathological processes including immune-mediated disorders, cancer and fibrosis^8–10^. Moreover, miRNAs have been found to be involved in regulation of differentiation and proliferation of specific immune subsets^11^.

The aim of the current study was to investigate the expression of a panel of miRNAs and cytokines in lung transplant recipients who received alemtuzumab induction therapy. Our hypothesis is that microRNA profiles in the two population of subjects might differ substantially, thus suggesting possible mechanistic effects of alemtuzumab induction therapy (Supplementary Informations 2, 3, 4, 5).

## Results

### Demographics

Sixty patients (30 vs. 30) were included in the analysis. Seven patients (4 in the control group and 3 in the alemtuzumab group) had to be excluded, since at least one microRNA was not detectable at one time point. Baseline characteristics were similar among the groups (Table 1). Median age was 58 years (IQR: 55–60) in the control group compared to 52 years (IQR: 46–59) in the alemtuzumab group. The most represented underlying diagnosis was chronic obstructive lung disease in both groups. Approximately 20% of recipients in both groups had a high risk cytomegalovirus (CMV) match (Donor − /Recipient +). Table 1 summarizes patient characteristics of both study groups.

**Table 1:** Group demographics.

Parameters		Groups		
		Controls (*n* = 26)	Alemtuzumab (*n* = 27)	*p*-value
Female (*n*, %)		14, 54%	15, 56%	0.692
Age (median, IQR)		58 (55–60)	52 (46–59)	0.520
Underlying diagnosis (*n*, %)	Obstructive diseases	22, 85%	16, 59%	0.163
	Fibrosis	2, 7%	5, 19%	
	PH	1, 4%	2, 7%	
	CF	0	3, 11%	
	Others	1, 4%	1, 4%	
CMV risk (*n*, %)	D + /R-	5, 19%	6, 22%	0.400
	D + /R +	9, 35%	12, 44%	
	D-/R +	8, 31%	4, 15%	
	D-/R-	4, 15%	5, 19%	
Bilateral lung transplantation (*n*, %)		26, 100%	27, 100%	–
PGD at 72 h (*n*, %)	0	20, 77%	19 (70%)	0.879
	1	4, 15%	4 (15%)	
	2	1, 4%	2 (7%)	
	3	0	0	
	Ungradable	1, 4%	2 (7%)	
High grade ACR (*n*, %)		7, 29%	0	0.003
High grade LB (*n*, %)		10, 42%	4, 15%	0.032
Cumulative A score (*n*, %)		0.11 (0–0.24)	0 (0–0.007)	0.002
Cumulative B score (*n*, %)		0.63 (0.39–0.92)	0.31 (0–14-0.59)	0.003

### Longitudinal miRNAs expression

For the present study, we selected thirteen miRNAs (miR-30b, miR-125a, miR-99a, miR-17, miR-23b, miR-98, miR-155, miR-182, miR-181a, miR-21, miR-24, miR-31 and miR-146a) involved in the development, proliferation and function of immune system. MiR-155 and miR-146 are probably the two most studied immuno-microRNAs, due to their regulatory role in different immune cell subsets. Moreover, both miR-155 and miR146a are associated with clinical operational tolerance in transplant recipients^12^. MiR-30b, miR-125a, miR-99a, miR-17 and miR-23b were previously demonstrate to be dysregulated in tolerogenic dendritic cells (DC) ^13^. MiR-155 exerts a central role in the maturation of B-cells and plasma cell commitment ^14–17^ and in the differentiation of T helper cells^18^. MiR-17–92 cluster was shown to regulate B-cell proliferation, development and immunoglobulin rearrangement by targeting Bim and PTEN ^19^. Moreover, miR-155, miR-146a, miR-182, miR-181a, miR-21, miR-24, miR-31 and miR-146a, were reported as involved in T cell regulation ^20–22^.

**Figure 1:**
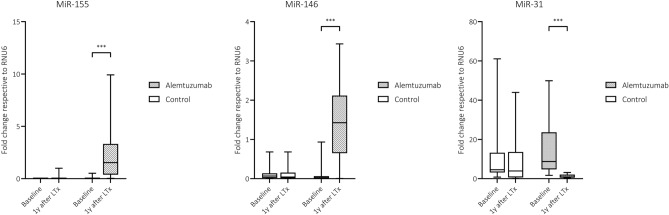
Expression difference of the analyzed dysregulated miRNAs at baseline and one year after induction therapy within the same groups. The Figure shows box-plots depicting miRNA expressions at the two timepoints. Both groups are represented on the graph next to each other. Mann–Whitney test was used to test the difference in expression between the two time points within the same group. In alemtuzumab group miR-155 and miR-146 are significantly upregulated compared to baseline levels. On the contrary, miR-31 is drastically downregulated. The boxes indicate interquartile range, the line across the boxes the median and the lower and upper margin of the vertical lines the minimum and maximum, respectively. *p = 0.05, **p < 0.01, *** *p* < 0.001.

The no-induction group did not show any change in expression of miRNAs overtime. Instead, patients who received alemtuzumab had significantly higher expression of miR-146a and miR-155 one year after transplantation (*p* < 0.001 and *p* < 0.001, respectively) (Table 2, Fig. 1). MiR-31 was found downregulated one year after transplantation in the alemtuzumab group (eightfold change, *p* < 0.001, Fig. 1).

**Table 2:** MicroRNA expression differences within the groups between the baseline and one year after transplantation.

Timepoint	miRNA	Groups						
		Baseline			One year after transplantation			*p*-value
		Median	Percentile 25	Percentile 75	Median	Percentile 25	Percentile 75	
No-induction	miR-30b	0.636	0.271	1.082	0.433	0.011	1.329	0.578
	miR-24	0.034	0.007	0.047	0.026	0.002	0.055	0.821
	miR-98	0.892	0.243	1.117	0.639	0.047	1.161	0.226
	miR-99a	6.664	2.874	12.910	5.819	0.310	13.320	0.680
	miR-23b	6.002	1.749	8.048	4.085	0.241	10.478	0.606
	miR-125a	0.309	0.013	0.520	0.178	0.016	0.456	1.000
	miR-182	0.009	0.002	0.011	0.005	0.002	0.015	0.342
	miR-17	0.039	0.013	0.044	0.018	0.001	0.053	0.216
	miR-486	0.190	0.138	0.228	0.089	0.015	0.287	0.312
	miR-21	0.590	0.144	1.712	0.463	0.027	1.055	0.578
	miR-146a	0.067	0.020	0.108	0.042	0.006	0.135	0.381
	miR-155	0.006	0.002	0.015	0.005	0.000	0.022	0.765
	miR-31	6.275	3.903	17.957	4.221	0.621	13.619	0.337
Alemtuzumab	miR-30b	0.171	0.007	0.562	0.202	0.069	1.168	0.684
	miR-24	0.010	0.001	0.028	0.009	0.003	0.039	0.802
	miR-98	0.445	0.023	0.990	0.557	0.151	1.641	0.522
	miR-99a	13.985	0.828	46.339	9.165	3.510	26.038	0.831
	miR-23b	6.930	0.633	26.653	8.007	2.694	40.027	0.373
	miR-125a	0.036	0.008	0.165	0.091	0.020	0.154	0.802
	miR-182	0.001	0.000	0.007	0.003	0.001	0.017	0.377
	miR-17	0.008	0.000	0.040	0.011	0.003	0.029	0.938
	miR-486	0.025	0.007	0.088	0.030	0.006	0.087	0.831
	miR-21	0.128	0.004	0.244	0.116	0.004	0.481	0.654
	**miR-146a**	**0.027**	**0.001**	**0.124**	**1.425**	**0.662**	**1.990**	**0.001**
	**miR-155**	**0.002**	**0.000**	**0.032**	**1.532**	**0.382**	**3.250**	**0.001**
	**miR-31**	**8.190**	**4.428**	**10.373**	**0.898**	**0.580**	**2.305**	**0.001**

Significant values are in [bold/italics].

### Comparison of miRNA expression between groups

**Figure 2:**
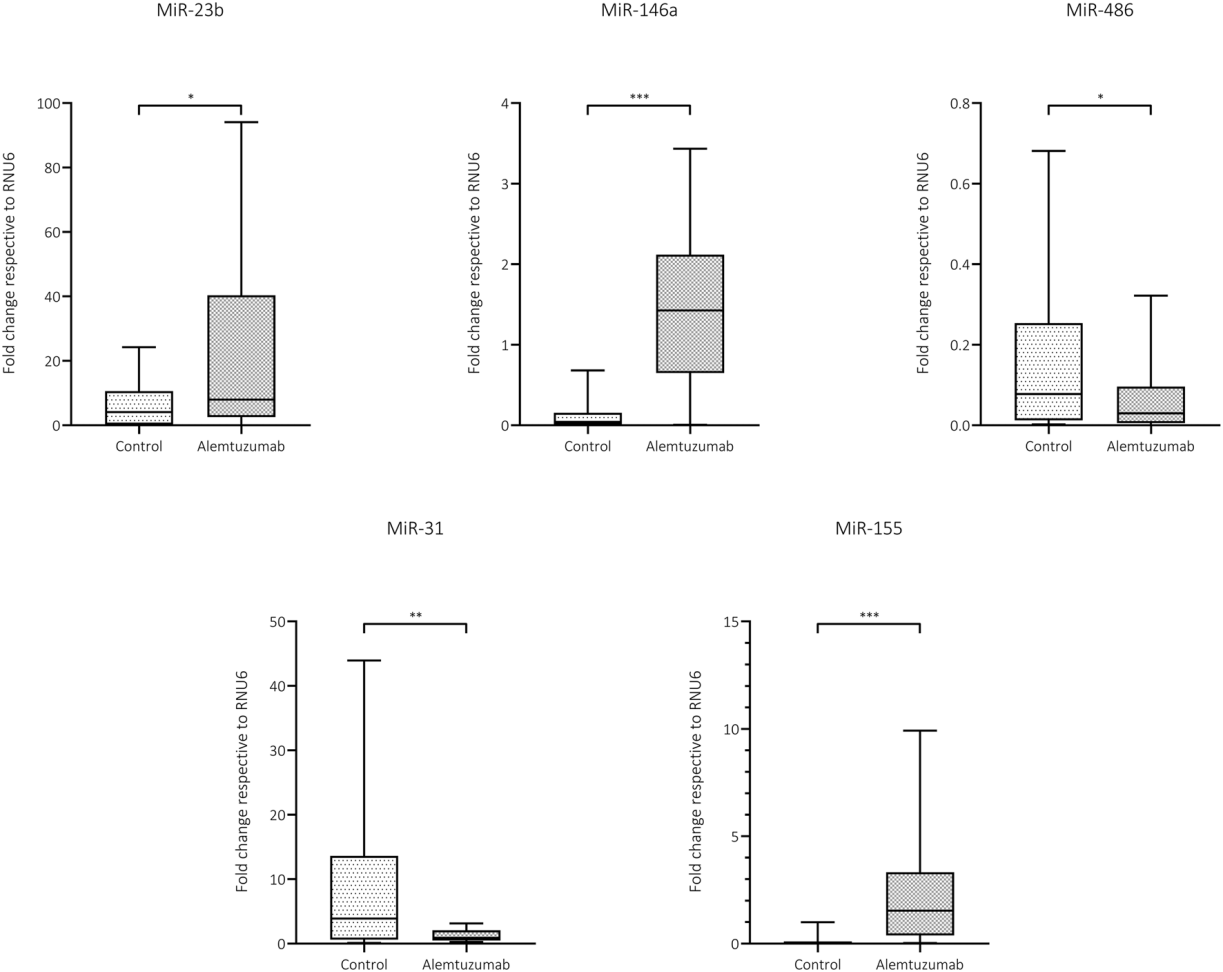
Expression difference of the analyzed dysregulated miRNAs between the two study groups at one year after induction therapy. The Figure shows box-plots depicting miRNA expressions of the two groups at the same timepoint, 12 months after induction therapy. Mann–Whitney test was used to test the difference in expression between the two groups at the same timepoint. MiR-155, miR-146 and miR-23b are significantly upregulated compared to no-induction group. On the contrary, miR-486 is mildly downregulated. The boxes indicate interquartile range, the line across the boxes the median and the lower and upper margin of the vertical lines the minimum and maximum, respectively. *p = 0.05, **p < 0.01, ***p < 0.001.

One year after transplantation, five miRNAs showed different expression levels among the groups: miR-23b, miR-486, miR-146a and miR-155 and miR-31 (Fig. 2, Table 3). MiR-23b was upregulated with a fourfold change in the control group and an eightfold change in the alemtuzumab group (*p* = 0.046). Both miR-146 and miR-155 were upregulated in alemtuzumab groups and downregulated in the no-induction group (*p* < 0.001 and *p* < 0.001, respectively). MiR-486 was dysregulated both at baseline and one year after transplantation, thus it is conceivable to hypothesize that the difference is not real (Table 3). MiR-31 was downregulated in the alemtuzumab group compared to the no-induction group (*p* = 0.025).

**Table 3:** MicroRNA expression differences between the groups at baseline and one year after transplantation.

Timepoint	miRNA	Groups						
		No-induction			Alemtuzumab			*p*-value
		Median	Percentile 25	Percentile 75	Median	Percentile 25	Percentile 75	
Baseline	miR-30b	0.636	0.271	1.082	0.171	0.007	0.562	0.097
	miR-24	0.034	0.007	0.047	0.010	0.001	0.028	0.215
	miR-98	0.892	0.243	1.117	0.445	0.023	0.990	0.288
	miR-99a	6.664	2.874	12.910	13.985	0.828	46.339	0.728
	miR-23b	6.002	1.749	8.048	6.930	0.633	26.653	0.636
	miR-125a	0.309	0.013	0.520	0.036	0.008	0.165	0.157
	miR-182	0.009	0.002	0.011	0.001	0.000	0.007	0.076
	miR-17	0.039	0.013	0.044	0.008	0.000	0.040	0.157
	**miR-486**	**0.190**	**0.138**	**0.228**	**0.025**	**0.007**	**0.088**	**0.016**
	miR-21	0.590	0.144	1.712	0.128	0.004	0.244	0.110
	miR-146a	0.067	0.020	0.108	0.027	0.001	0.124	0.475
	miR-155	0.006	0.002	0.015	0.002	0.000	0.032	0.875
	miR-31	6.275	3.903	17.957	8.190	4.428	10.373	0.928
One year after transplantation	miR-30b	0.433	0.011	1.329	0.202	0.069	1.168	0.450
	miR-24	0.026	0.002	0.055	0.009	0.003	0.039	0.234
	miR-98	0.639	0.047	1.161	0.557	0.151	1.641	0.831
	miR-99a	5.819	0.310	13.320	9.165	3.510	26.038	0.161
	**miR-23b**	**4.085**	**0.241**	**10.478**	**8.007**	**2.694**	**40.027**	**0.046**
	miR-125a	0.178	0.016	0.456	0.091	0.020	0.154	0.122
	miR-182	0.005	0.002	0.015	0.003	0.001	0.017	0.629
	miR-17	0.018	0.001	0.053	0.011	0.003	0.029	0.299
	**miR-486**	**0.089**	**0.015**	**0.287**	**0.030**	**0.006**	**0.087**	**0.036**
	miR-21	0.463	0.027	1.055	0.116	0.004	0.481	0.120
	**miR-146a**	**0.042**	**0.006**	**0.135**	**1.425**	**0.662**	**1.990**	**0.000**
	**miR-155**	**0.005**	**0.000**	**0.022**	**1.532**	**0.382**	**3.250**	**0.000**
	**miR-31**	**3.890**	**0.621**	**13.619**	**0.898**	**0.580**	**1.850**	**0.025**

Significant values are in [bold/italics].

### Cytokine levels

**Figure 3:**
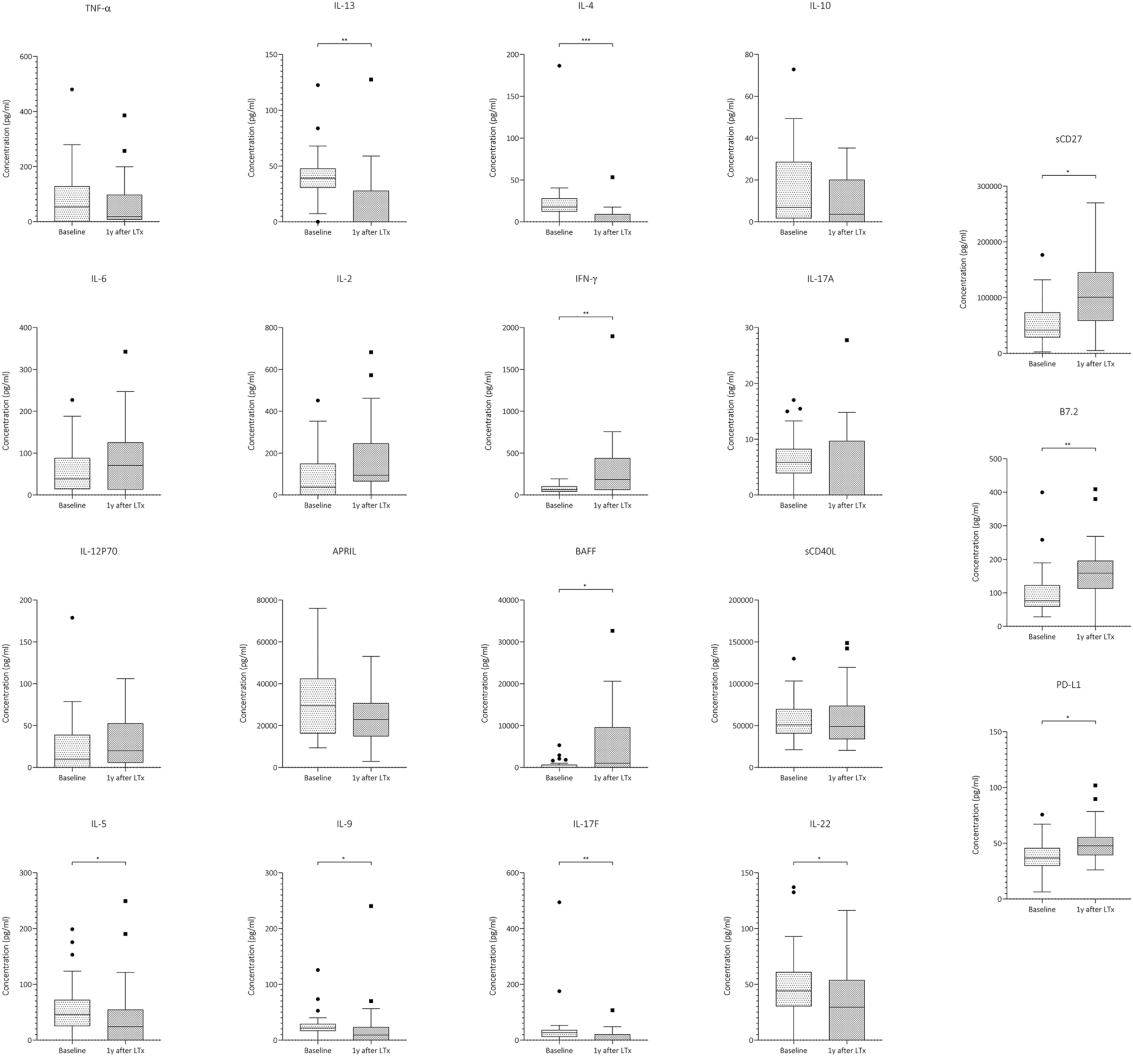
Expression difference of the measured cytokines within control group at baseline and one year after induction therapy. The Figure shows box-plots depicting cytokine expressions at the two timepoints measured with flow cytometry. IL-13, IL-4, IL-5, IL-9, IL-17F, IL-22 are significantly downregulated compared to baseline levels. Interferon-γ, B cell activating factor (BAFF), sCD27, B7.2 and PD-L1 are significantly upregulated. The boxes indicate interquartile range, the line across the boxes the median and the lower and upper margin of the vertical lines the minimum and maximum, respectively. *p = 0.05, **p < 0.01, ***p < 0.001.

In no-induction group, **IL-13** (39 pg/ml, IQR 31–48 vs. 0.1 pg/ml, IQR 0.1–28, *p* = 0.002), **IL-4** (18 pg/ml, IQR 13–28 vs. 0.1 pg/ml, IQR 9–53, *p* < 0.001), **IL-5** (46 pg/ml, IQR 25–72 vs. 24 pg/ml, IQR 0.1–54, *p* = 0.031), **IL-9** (22 pg/ml, IQR 17–29 vs. 9 pg/ml, IQR 0.1–23, *p* = 0.006), **IL-17F** (27 pg/ml, IQR 12–36 vs. 0.1 pg/ml, IQR 0.1–21, *p* = 0.001) and **IL-22** (44 pg/ml, IQR 31–61 vs. 30 pg/ml, IQR 0.1–54, *p* = 0.033) showed lower levels one year after transplantation compared to baseline (Fig. 3, Table S1). On the contrary, the concentration of **IFN-γ** (65 pg/ml, IQR 40–102 vs. 183 pg/ml, IQR 62–440, *p* = 0.003) and B-cell activating factor **(BAFF)** (0.1 pg/ml, IQR 0.1–628 vs. 998 pg/ml, IQR 0.1–9586, *p* = 0.008) increased overtime. Similarly, in this group, **sCD27** (38,161 pg/ml, IQR 29,532–70,727 vs. 91,385 pg/ml, IQR 67,772–139,278, *p* = 0.026), **B7.2** (66 pg/ml, IQR 59–96 vs. 152 pg/ml, IQR 115–192, *p* = 0.001) and **sPD-L1** (38 pg/ml, IQR 31–44 vs. 48 pg/ml, IQR 37–55, *p* = 0.042) showed higher concentration levels at one-year post-transplant (Table S3, Fig. 3).

**Figure 4:**
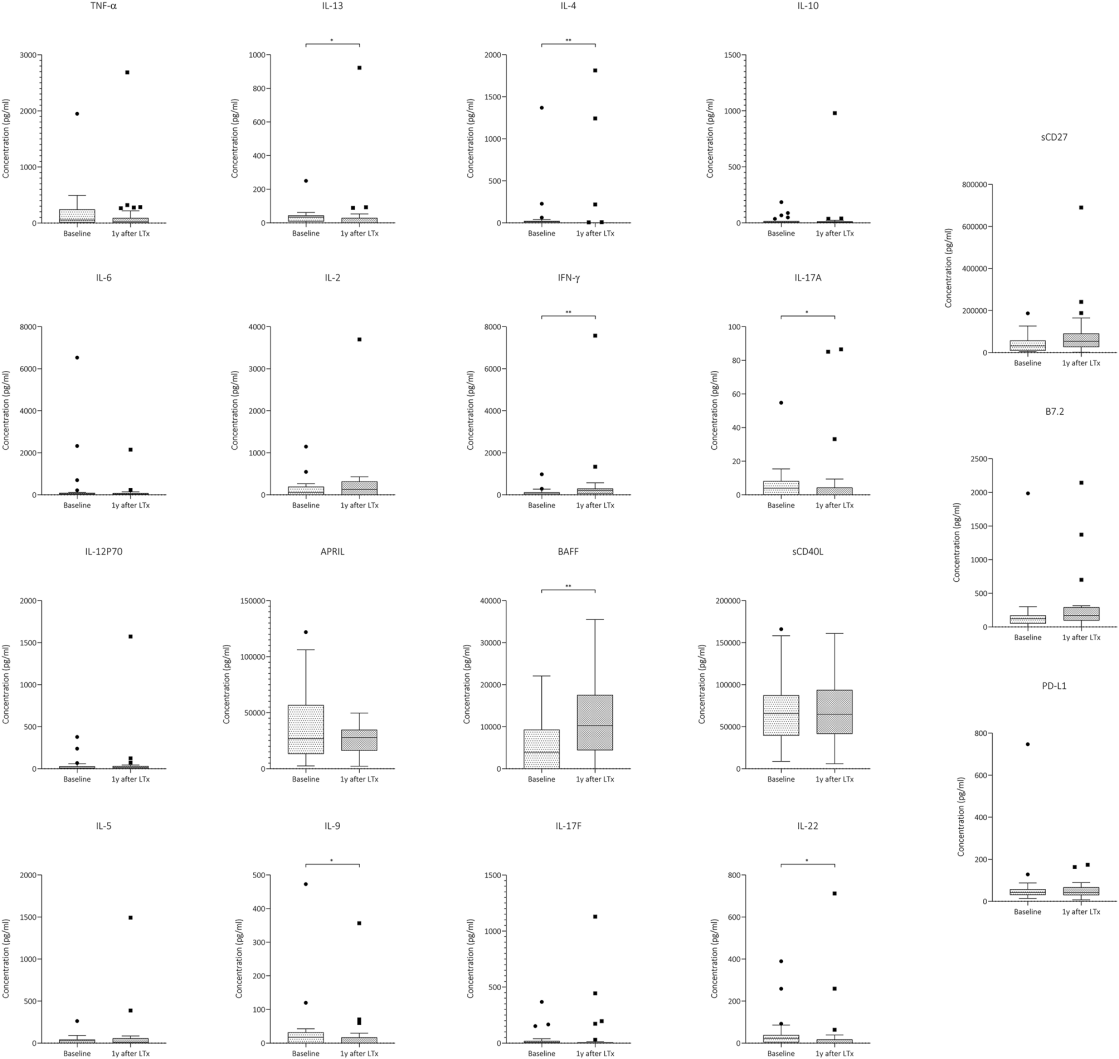
Expression difference of the measured cytokines within alemtuzumab group at baseline and one year after induction therapy. The Figure shows box-plots depicting cytokine expressions at the two timepoints measured with flow cytometry. IL-13, IL-4, IL-17A, IL-9, IL-22 are significantly downregulated compared to baseline levels. Interferon-γ and B cell activating factor (BAFF) are significantly upregulated. The boxes indicate interquartile range, the line across the boxes the median and the lower and upper margin of the vertical lines the minimum and maximum, respectively. *p = 0.05, **p < 0.01, ***p < 0.001.

Patients who received alemtuzumab had lower serum levels of **IL-13** (34 pg/ml, IQR 9–43 vs. 0.1 pg/ml, IQR 0.1–29, *p* = 0.041), **IL-4** (12 pg/ml, IQR 0.1–20 vs 0.1 pg/ml, IQR 0.1–0.1, *p* = 0.005), **IL-17A** (4 pg/ml, IQR 0.1–8 vs. 0.1 pg/ml, IQR 0.1–4, *p* = 0.043), **IL-9** (17 pg/ml, IQR 0.1–32 vs. 0.1 pg/ml, IQR 0.1–17, *p* = 0.026) and **IL-22** (23 pg/ml, IQR 4–38 vs. 0.1 pg/ml, IQR 0.1–17, *p* = 0.015) one year after transplantation (Fig. 4) with respect to baseline. Instead, levels of BA**FF** (3934 pg/ml, IQR 0.1–9270 vs. 10,232 pg/ml, IQR 4436–17,518, *p* = 0.015) and **IFN-γ** (23 pg/ml, IQR 4–38 vs. 0.1 pg/ml, IQR 0.1–17, *p* = 0.015) increased overtime (Table S1).

At baseline, median BAFF serum level was higher in alemtuzumab group than in no-induction group (3934 pg/ml, IQR: 0.1–9270 vs. 0.1 pg/ml, IQR: 0.1–628, *p* = 0.001), while median serum levels of IL-5 (28.1 pg/ml, IQR: 0.1–40.2 vs. 45.7 pg/ml, IQR: 25.4–71.9), IL-17F (7.2 pg/ml, IQR: 0.1–17.6 vs. 26.8 pg/ml, IQR: 12.3–36) and IL-22 (22.8 pg/ml, IQR: 3.9–38 vs. 44.2 pg/ml, IQR: 30.5–60.9) were higher in no-induction group (*p* = 0.019, *p* = 0.002, *p* = 0.008, respectively). At one year after transplantation, median BAFF serum levels remained significantly higher in alemtuzumab group (10,232 pg/ml, IQR: 4436–17,518 vs. 998 pg/ml, IQR: 0.1–9586, *p* = 0.011), while only IL-22 showed constantly higher values in no-induction group (0.1 pg/ml, IQR: 0.1–16.5 vs. 29.5 pg/ml, IQR: 0.1–53.7, *p* = 0.018) (Table S2).

### Correlations

One year after transplantation, BAFF positively correlated with miR-146a (r = 0.288, *p* = 0.040) and miR-155 (r = 0.261, *p* = 0.050) while IL-17A negatively correlated with miR-155 (r = 0.418, *p* = 0.033) in alemtuzumab group (Figure S5). No other significant correlations were observed.

### MiRNA gene target prediction and pathway enrichment analysis

After identification of dysregulated miRNAs, gene target prediction analysis was performed using miR-155, miR-146a, miR-23b and miR-31. The total number of identified gene targets after accounting for overlap was 10,461. Among the identified genes, *IL-17A* and *TNFSF13B* genes, which encode IL-17A and BAFF respectively, were found to be targets of the dysregulated miRNAs. Using the identified gene targets, we performed enrichment analysis to identify significantly enriched pathways using the Kyoto Encyclopedia of Genes and Genomes (KEGG) database. MultiMir revealed 39 significantly enriched pathways with a *p*-value < 0.05 and a false discovery rate (FDR) < 0.10 (Benjamini–Hochberg procedure) in alemtuzumab group compared to controls at one year after transplantation. Similarly, 20 significantly enriched pathways were identified at one year after transplantation compared to baseline in alemtuzumab patients. The pathway names, odds ratios and levels of significance are listed in Tables S4 and S5. Analysis of the identified gene targets showed that signaling pathways involved in immune cell proliferation were enriched: Toll-like receptor (TLRs) signaling pathway, cytokine downstream signaling pathways, mammalian target of rapamycin (mTOR) pathway, Janus kinase (JAK)-Signal Transducers and Activators of Transcription (STAT) signaling pathway, mitogen-activated protein kinase (MAPK) pathway and PI3K-Akt signaling pathway (Tables S3 and S4). Moreover, transcription factors fundamental in immune cell activation such as forkhead box protein O1 (FOXO1), signal transducer and activator of transcription 1 and 5 (STAT1 and STAT 5), suppressor of cytokine signaling 1 (SOCS 1), nuclear factor kappa B (NF-kB), TNF receptor associated factor 6 (TRAF 6), interleukin-1 receptor-associated kinase 1 (IRAK 1), bHLH transcription factor (c-Myc) and SMAD family members were found as main targets of the differentially regulated miRNAs.

## Discussion

Over the last 20 years, constant refinements of the immunosuppression protocols contributed to the improvement of long-term outcome after lung transplantation. Alemtuzumab is a monoclonal antibody, recently used as induction agent in solid organ transplantation. Based on the published experience, this agent shows excellent results in terms of rejection rates and survival^4–6,23^. MiRNAs are post-transcriptional regulators of complex biological and pathological processes and have been identified to exert a key function in hematopoiesis and immune cell homeostasis and activation. In the current study, we identified a set of differentially regulated miRNAs (miR-155, miR-146a, miR-23b, miR-31 and miR-486) one year after alemtuzumab induction therapy. Moreover, a panel of cytokines were found to be differently in both no-induction and alemtuzumab. Only in the alemtuzumab group, BAFF and IL-17A showed a positive and negative correlations with miR-146 and miR-155, respectively. Finally, enrichment pathway analysis supported the hypothesis that these five miRNAs are key regulators of immune cell proliferation, lineage commitment and inflammatory response.

Alemtuzumab is a monoclonal antibody against CD52, which is a glycoprotein found on the surface of lymphocytes, monocytes and dendritic cells. The engagement of alemtuzumab on CD52 causes a profound long-lasting immunodepletion ^2,3^. B cell counts recover within 3–6 months and T cell counts within 12–24 months after treatment ^2,24^. After reconstitution, phenotypic changes in the immune cell subsets seem to play a crucial role in allograft tolerance mechanisms ^2,24–30^.

MicroRNAs are known key players in the pathogenesis of autoimmune diseases, fibrosis and cancer but their role in the context of immunosuppression after lung transplantation has not been studied yet. For the first time, could show herein that miR-155 and miR-146a, two key orchestrators of hematopoiesis and immune cell function are upregulated one year after alemtuzumab treatment. MiR-155 controls T-cell lineage commitment, by promoting T helper type 1 (Th1) over T helper type 2 (Th2) cells^14^. It has been previously described that deficiency of miR-155 leads to decreased germinal center (GC) size and response, lower levels of plasma and memory cells and impaired production of high-affinity class-switched IgGs^14,31,32^. MiR-146a is dynamically regulated during CD4^+^ cell differentiation, with an upregulation in differentiated Th1 and a downregulation in Th2 cells^33^. Thus, it is reasonable to hypothesize that both miRNAs may actively shape the reconstitution of immune cell subsets observed after alemtuzumab treatment^24,27–29^.

B-cell activating factor is a cytokine belonging to TNF family, emerged as a critical factor for B cell survival and maturation^34^. Three main receptors of BAFF are known: B-cell maturation antigen (BCMA), Transmembrane activator and CAML interactor (TACI) and BAFF-R. BAFF binds strongly to TACI and BAFF-R but only weakly with BCMA^35^. BAFF-R is critical for survival and maturation of B cell precursors, it is expressed at late transitional stage and is present on all mature B cells^36^. TACI is present on late transitional stage and BCMA is present on plasma cells and is responsible for their long-lived survival^37–39^. The cytokine A proliferation-inducing ligand (APRIL) shares the receptor BCMA and TACI with BAFF, however, contrarily to BAFF, it strongly binds to BCMA and weakly to TACI. This slightly different expression pattern independently regulates homeostatic proliferation of different B-cell subsets^40^. In our cohort, we observed a strong increase in BAFF expression but not of APRIL. Concomitantly, serum levels of sCD40L and IL-6, which are both required for B cell maturation in plasma cells and IL-6 production^41,42^, did not increase overtime. Thus, the isolated increased expression of BAFF may indicate the proliferation of memory B cells, a cell subset which is spared by alemtuzumab^2,24^. Similar to our findings, serum BAFF levels but not APRIL were found to be increased in a cohort of kidney recipients after alemtuzumab induction^43^*.* We found a positive correlation between miR-155 and BAFF. A similar finding was observed in rheumatoid arthritis patients, whose CD19^+^ B cells expressed miR-155 under stimulation by BAFF^44^. As miR-155 plays a critical role in early B cell commitment and proliferation, it is reasonable to hypothesize that, after alemtuzumab immunodepletion, the memory B cells compartment undergoes a homeostatic proliferation driven by miR-155 through the mediation of BAFF. However, this mechanism needs to be further clarified in future experiments.

In the current study, miR-31 was found to be significantly down-regulated in alemtuzumab patients. It is well described that miR-31 positively correlates with T cell activation and induces the transcription of IL-2 in T cells by suppression of RhoA and activation of nuclear factor of activated T-cells (NF-AT)^45,46^. Moreover, miR-31 directly targets IL-25 transcription, with the consequent production of IL-12 and IL-23 by antigen-presenting cells and an increased Th1/Th17 response^47^. Thus, downregulation of miR-31 may significantly contribute to contain T cell activation. An analogous effect can be ascribed to the upregulation of miR-146a, an anti-inflammatory microRNA, which we found in the alemtuzumab group. Engagement of T-cell receptor (TCR) triggers expression of miR-146a which negatively targets NF-kB and inflammatory response ^48^. Taken together, it is reasonable to hypothesize that the downregulation of miR-31 and upregulation of miR-146 might participate in the regulation of T cell responses and the Tregs/Th17 balance, thus favoring graft tolerance. Alemtuzumab, in fact, has been associated with an increase in peripheral levels of anti-inflammatory cytokines and with a suppression of Th1 and Th17 transcription factors^25,26^. This is further supported in our findings by the negative correlation found between miR-155 and IL-17A.

The frequency of Treg cells seems to increase after alemtuzumab immunodepletion^24,27,28^. MicroRNAs have been found to be fundamental regulators of Tregs commitment and proliferation^49^. MiR-155 binds to the promoter region of FoxP3^50^. Increased FoxP3 transcription induces miR-155 expression, which further stimulates Tregs proliferation in a feedback loop^51^. Rudensky’s group also showed that miR-146 is upregulated in Tregs^52^. MiR-31 is known to target FoxP3 mRNA and negatively influence its transcription^53^, on the contrary miR-23 is a FoxP3 transcriptional target and its expression is involved in Tregs commitment^54^. This is in line with our observation of an upregulation of pro-tolerogenic miR-155/-146/-23b and downregulation of anti-tolerogenic miR-31 in the alemtuzumab group at 1 year after transplantation.

As ancillary finding, serum levels of three soluble co-signaling molecules, namely sCD27, B7.2 and sPD-L1, were increased one year after transplantation in no-induction group while remained unchanged in alemtuzumab group. Soluble CD27 is secreted by activated T-cells and plays a role in their maturation and proliferation. Increased levels have been detected in inflammatory and autoimmune diseases, viral infections and lymphoid malignancies, making it a potential marker for T-cell–mediated inflammation^55^. Similarly, soluble B7.2 is a costimulatory molecule of human T cells^56^ and it has been found increased in systemic lupus erythematosus, asthma and leukemia^57–59^. It is reasonable to hypothesize that soluble B7.2 could have a similar stimulatory effect in no-induction group. Finally, higher levels of sPD-L1 were associated with poorer long-term graft outcome in kidney recipients^60^. The soluble form of this molecule may play as antagonist of PD-1/PD-L1 engagement, thereby stimulating proliferation and cytokine production of T cells^61^. Serum levels of a set of cytokines have been found significantly different one year after transplantation compared to baseline. IL-4, IL-13, IL-5, IL-9 and IL-22 are all reduced one year after transplantation. This finding is most likely due to the suppressing effects of the maintenance immunosuppression protocol (mainly CNI and steroids) on T cell activation. On the contrary, IFN-γ, a critical cytokine for the innate and adaptive immunity, is increased in both groups. This cytokine is produced mainly by NK and NKT cells and by CD4^+^ and CD8^+^, it is, therefore, reasonable to hypothesize that the increase of IFN-γ is associated to the antigen-specific immune response.

Our study is not free of limitations. First, quantification of immune cell subsets by FACS is lacking. This could have strengthened our results, by correlating the presented molecular signature with the cellular findings^2,24^. Thus, we can only speculate on which immune cells are responsible for the observed miRNA dysregulation. Further, serum samples beyond one year after transplantation were not available for analysis. This would have deepened our knowledge about the repopulation of other immune cell subsets as well as the possible long-term effects of induction therapy. Furthermore, the explanatory and pilot nature of the study may solely suggest possible underlying processes, which have to be further confirmed by mechanistic studies. On the other side, it should be acknowledged that the current analysis represents the first study describing different miRNAs expression after LTx in a prospectively collected cohort after alemtuzumab induction.

In conclusion, this report provides the first evidence of miRNAs and cytokine dysregulation following alemtuzumab induction therapy in lung transplantation. Since these dysregulated mediators play a role in the homeostatic reconstitution of different B cell subsets and in the regulation of T cell response, their role in regulation of graft tolerance and in humoral complications can be envisaged. Further research, however, is necessary to correlate their expression with the activation of specific molecular pathways in different T and B cell subsets in order to increase our knowledge and ultimately improve the efficacy of our immunosuppressive strategies.

## Methods

### Study design

This is a comparative study, including serum samples of lung transplant recipients prospectively collected between June 2008 and December 2013 at two timepoints: time of transplantation and 12 months post-transplant. Within this period a total of 710 lung transplant recipients consented for prospective storage of biological samples including serum, plasma or bronchoalveolar lavage (BAL) for scientific purposes. Written informed consent was obtained from all recipients. Among them, 252 received alemtuzumab as induction therapy and for 30 patients, serum samples for the two timepoints defined by the study protocol were available. The remaining 222 patients could not be included in the analysis for the following reasons: (1) they did not have serum samples at both timepoints, (2) the serum samples were obtained at day of listing, (3) the collected biological samples did not include serum. The 30 control patients were chosen among the 233 patients who did not receive any induction therapy and had serum samples at both timepoints. Control patients were matched based on the following variables: gender, age, underlying diagnosis, CMV risk, type of transplantation and primary graft dysfunction (PGD) grade (Table 4).

**Table 4:** Maintenance immunosuppression protocol.

Time after Tx	Tacrolimus (target trough level)		Steroids		Mycophenolate mofetil	
	No induction	Alemtuzumab	No induction	Alemtuzumab	No induction	Alemtuzumab
0–3 months	15–18 ng/ml	10–12 ng/ml	0.3 mg/kg	0.2 mg/kg	1–1,5 g twice a day	–
3–6 months	13–15 ng/ml	8–10 ng/ml	0.2 mg/kg	0.15 mg/kg	1–1,5 g twice a day	–
6–12 months	10–12 ng/ml	6–8 ng/ml	0.15 mg/kg	0.1 mg/kg	1–1,5 g twice a day	–
12–24 months	8–10 ng/ml	5–7 ng/ml	5 mg/d	5 mg/d	1–1,5 g twice a day	1–1,5 g twice a day

Inclusion criteria were primary lung transplantation, adult age and availability of serum samples both at time of transplantation and 1 year after transplantation. Exclusion criteria were multi-organ transplantation, retransplantations and pediatric age. No patient had signs or diagnosis of acute cellular rejection, antibody-mediated rejection, infection or CLAD at time of blood sampling. All experiments and methods were performed in accordance with relevant guidelines and regulations. The study has been approved by the Institutional Ethical Committee of the Medical University of Vienna [ECS 1729/2020] and was conducted according the declaration of Helsinki.

### Clinical protocol and definitions

Lung transplant recipients received either alemtuzumab (Genzyme/Sanofi, Cambridge, USA) as a single intravenous dose of 30 mg after LTx at arrival at the intensive care unit (ICU) or no induction therapy. Recipients after alemtuzumab induction received a low-dose CNI-based maintenance immunosuppression, while recipients without induction therapy received a triple-drug protocol (IS protocols and target blood levels are shown in Table 1). During follow-up, in case of deterioration of kidney function, infections requiring hospital admission or rejection, the target blood levels were adapted to the low or high end of the respective target range (Table 1). Perioperative infectious prophylaxis was based on broad-spectrum antibiotics or adapted to resistance testing. All patients received a lifelong *Pneumocystis* prophylaxis with trimethoprim-sulfamethoxazole or atovaquon. Prophylactic inhalation therapy with amphotericin B and gentamycin or according to pretransplant airway colonization was provided for 1–3 months. CMV prophylaxis included CMV hyperimmunoglobulines (POD 1,7,14 and 21) together with valganciclovir for a minimum of 3 months. In high-risk patients (donor + /recipient −) a 12 months prophylaxis was performed. Surveillance bronchoscopy with transbronchial biopsy (TBB) and BAL were performed 2 weeks and 1, 2, 3, 6, 12 months after transplantation and whenever clinically indicated. All patients received a chest computed tomography (CT) once a year. Biopsies were classified according to ISHLT criteria ^62^. ACR grade A2 and LB grade B2 or higher were treated with a pulse of steroids for 3 days with consecutive dose tapering. In case of inadequate clinical response, ATG (2 mg/kg) was administered for 5 days.

### Quantitative real-time PCR

We analyzed the expression levels of 13 miRNAs: hsa-miR-30b-5p, hsa-miR-24-3p, hsa-miR-98-5p, hsa-miR-99a-5p, hsa-miR-23b-3p, hsa-miR-125a-5p, hsa-miR-182-5p, hsa-miR-17-5p, hsa-miR-486-5p, hsa-miR-21-5p, hsa-miR-146a-5p, hsa-miR-155-5p and hsa-miR-31-5p. These miRNAs have been selected because previously found involved in regulating the proliferation and function of main immune cell types of the innate and adaptive immunity as well as in the pathogenesis of allograft rejection. Total RNA was isolated from serum samples using miRNeasy Serum/Plasma Kit (Qiagen). RNA concentration and purity were evaluated using a spectrophotometer. Total RNA was then reverse transcribed with the miRCURY LNA RT Kit (Qiagen) according to the manufacturer’s instructions. Quantitative real-time PCR (qRT-PCR) was performed with LNA probes (miRCURY LNA miRNA PCR Assay, Qiagen) followed by miRCURY SYBR Green PCR Kit (Qiagen) for all miRNAs. All miRNAs were detected using LNA Probes, which are high-affinity RNA analogs that hybridize to complementary miRNA sequences. miRCURY SYBR Green PCR Kit has been used to detect hybridized double-stranded sequence during PCR reaction. Expression levels of RNU6 were used as the normalization control. In order to identify the most robust candidate reference gene, stability values of selected miRNAs (hsa-miR-30b-5p, hsa-miR-24-3p, hsa-miR-98-5p, hsa-miR-99a-5p, hsa-miR-23b-3p, hsa-miR-125a-5p, hsa-miR-182-5p, hsa-miR-17-5p, hsa-miR-486-5p, hsa-miR-21-5p, hsa-miR-146a-5p, hsa-miR-155-5p, hsa-miR-31-5p and RNU6) were calculated and analyzed with NormFinder^63^. NormFinder identified RNU6 as the most stable reference genes, with a stability value of 0.056 (Table S6). All reactions were performed on an LC480 Real-Time PCR system (Roche Diagnostics) according to the manufacturer’s recommendations. Each experiment was performed in triplicates. The threshold cycle (Ct) was defined as the fraction cycle number at which fluorescence exceeded the given threshold. Relative gene expression level quantification was calculated using the 2^-ΔΔCt^ method.

### Cytokine expression by flow cytometry

A total of 17 cytokines were isolated from 1:1 diluted serum samples using the LEGENDplexTM Human Th Cytokine Panel (12-plex), the LEGENDplexTM Human B Cell Panel (13-plex) and Human Immune Checkpoint Panel 1 (12-plex) as per the manufacturer's instruction. These panels are bead-based multiplex assays, which use fluorescence-encoded beads. Quantification was performed using BD LSRFortessa™ Cell Analyzer. Each patients’ sample was measured in duplicates. Bead fluorescence was analyzed using LEGENDplexTM Software, which allows quantification of cytokines in pg/ml.

### In-silico data analysis

KEGG pathway enrichment analysis was performed using R version 4.0.5^64–66^. Genes targeted by miRNAs were identified using the *multiMiR* package. First, the “*get_multimir”* function was used to retrieve all targeted genes. The analysis was limited to experimentally validated miRNA-target interactions. MiRNA-target pairs with support type “negative” were not considered for further analysis. Next, the remaining target genes were mapped to KEGG pathways using the R package “*org.Hs.eg.db”*. Finally, pathways significantly enriched in genes targeted by miRNAs were identified using Fisher’s exact test. Across all performed tests, p-values were corrected for multiple testing using the “fdr” approach. For visualization purposes, significantly enriched pathways sharing at least 10 genes were connected, and the resulting graphs were plotted using the R package “*ggraph*”.

### Statistical analysis

Categorical variables were reported as absolute and relative frequencies (%), continuous variables as median (interquartile range, IQR) or mean (± standard deviation). Relative frequencies were calculated based on the number of patients alive in follow-up at the respective timepoint. Chi-square tests, Fisher exact tests, Mann–Whitney U-tests, or ANOVA were used to compare variables as applicable. Correlations were calculated using Spearman’s correlation test. Data was analyzed using SPSS version 26.0 software or R 4.0.5 and graphics were designed with GraphPad Prism 6.

## Supplementary Information

Supplementary Legends.

Supplementary Information 2.

Supplementary Information 3.

Supplementary Information 4.

Supplementary Information 5.

Supplementary Information 6.

Supplementary Information 7.

## Abbreviations

ACR: Acute cellular rejection
ATG: Anti-thymocyte globulin
BAFF: B-cell activating factor
BAL: Bronchoalveolar lavage
BCMA: B-cell maturation antigen
CD: Cluster of differentiation
CF: Cystic fibrosis
CLAD: Chronic lung allograft dysfunction
CMV: Cytomegalovirus
FOXO1: Forkhead box protein O1
IQR: Interquartile range
IRAK: Interleukin-1 receptor-associated kinase
IS: Immunosuppression
KEGG: Kyoto Encyclopedia of Genes and Genomes
LB: Lymphocytic bronchiolitis
LTx: Lung transplantation
MiRNA: Micro-RNA
NF-Kb: Nuclear factor kappa B
PCR: Polymerase chain reaction
PGD: Primary graft dysfunction
PH: Pulmonary hypertension
SOCS: Suppressor of cytokine signaling
STAT: Signal transducer and activator of transcription
TACI: Transmembrane activator and CAML interactor
TRAF: TNF receptor associated factor
