# Supplementary material for: Differential expression of circulating miRNAs after alemtuzumab induction therapy in lung transplantation
Source: Sci Rep. 2022 Apr 30;12:7072. doi: 10.1038/s41598-022-10866-w (PMC9056512; doi:10.1038/s41598-022-10866-w)
Supplement: Supplementary file 1 — Supplementary Legends. [file 41598_2022_10866_MOESM1_ESM.docx]

**Supplementary Figure legend**

**Figure S1 – Enrichment map showing enriched pathways organized into network of alemtuzumab versus no-induction.** The Figure shows the enrichment map organizing enriched terms into a network with edges connecting overlapping gene sets. In the enrichment map the enriched pathways found for the comparison of alemtuzumab patients against no-induction patients one year after transplantation. Pathways were included if they showed a false discovery rate < 0.1 and a connection was established if at least 10 gene were common in the connected pathways. The Figure was fully created by the authors using data from KEGG database^1-3^.

**Figure S2- Enrichment map showing enriched pathways organized into network of baseline versus 1 year after transplantation within alemtuzumab group.** The Figure shows the enrichment map organizing enriched terms into a network with edges connecting overlapping gene sets. In the enrichment map the enriched pathways found for the comparison of alemtuzumab patients against no-induction patients one year after transplantation. Pathways were included if they showed a false discovery rate < 0.1 and a connection was established if at least 10 gene were common in the connected pathways. The Figure was fully created by the authors using data from KEGG database^1-3^

**Figure S3 - Expression difference of the analyzed miRNAs at baseline and one year after induction therapy within the same groups.** The Figure shows box-plots depicting miRNA expressions at the two timepoints. Both groups are represented on the graph next to each other. Mann-Whitney test was used to test the difference in expression between the two time points within the same group. No expression difference was observed between the depicted miRNAs. The boxes indicate interquartile range, the line across the boxes the median and the lower and upper margin of the vertical lines the minimum and maximum, respectively.

**Figure S4 - Expression difference of the analyzed miRNAs between the two study groups at one year after induction therapy.** The Figure shows box-plots depicting miRNA expressions of the two groups at the same timepoint, 12 months after induction therapy. Mann-Whitney test was used to test the difference in expression between the two groups at the same timepoint. No expression difference was observed between the depicted miRNAs. On the contrary, miR-486 is mildly downregulated. The boxes indicate interquartile range, the line across the boxes the median and the lower and upper margin of the vertical lines the minimum and maximum.

**Figure S5 – Correlation plots analyzed between BAFF and miR-155 or miR-146a and IL-17A and miR-155.** One year after transplantation, A) BAFF positively correlated with miR-155 (r=0.261, p=0.050) and B) miR-146a (r=0.288, p=0.040), while C) IL-17A negatively correlated with miR-155 (r=0.418, p=0.033) in alemtuzumab group. No other significant correlations were observed. Correlations were calculated using Spearman’s correlation test.

1. Kanehisa M, Furumichi M, Sato Y, Ishiguro-Watanabe M, Tanabe M. KEGG: integrating viruses and cellular organisms. *Nucleic Acids Res.* 2021;49(D1):D545-d551.

2. Kanehisa M. Toward understanding the origin and evolution of cellular organisms. *Protein Sci.* 2019;28(11):1947-1951.

3. Kanehisa M, Goto S. KEGG: kyoto encyclopedia of genes and genomes. *Nucleic Acids Res.* 2000;28(1):27-30.
