# Supplementary figures and images for: Differential expression of circulating miRNAs after alemtuzumab induction therapy in lung transplantation
Source: Sci Rep. 2022 Apr 30;12:7072. doi: 10.1038/s41598-022-10866-w (PMC9056512; doi:10.1038/s41598-022-10866-w)

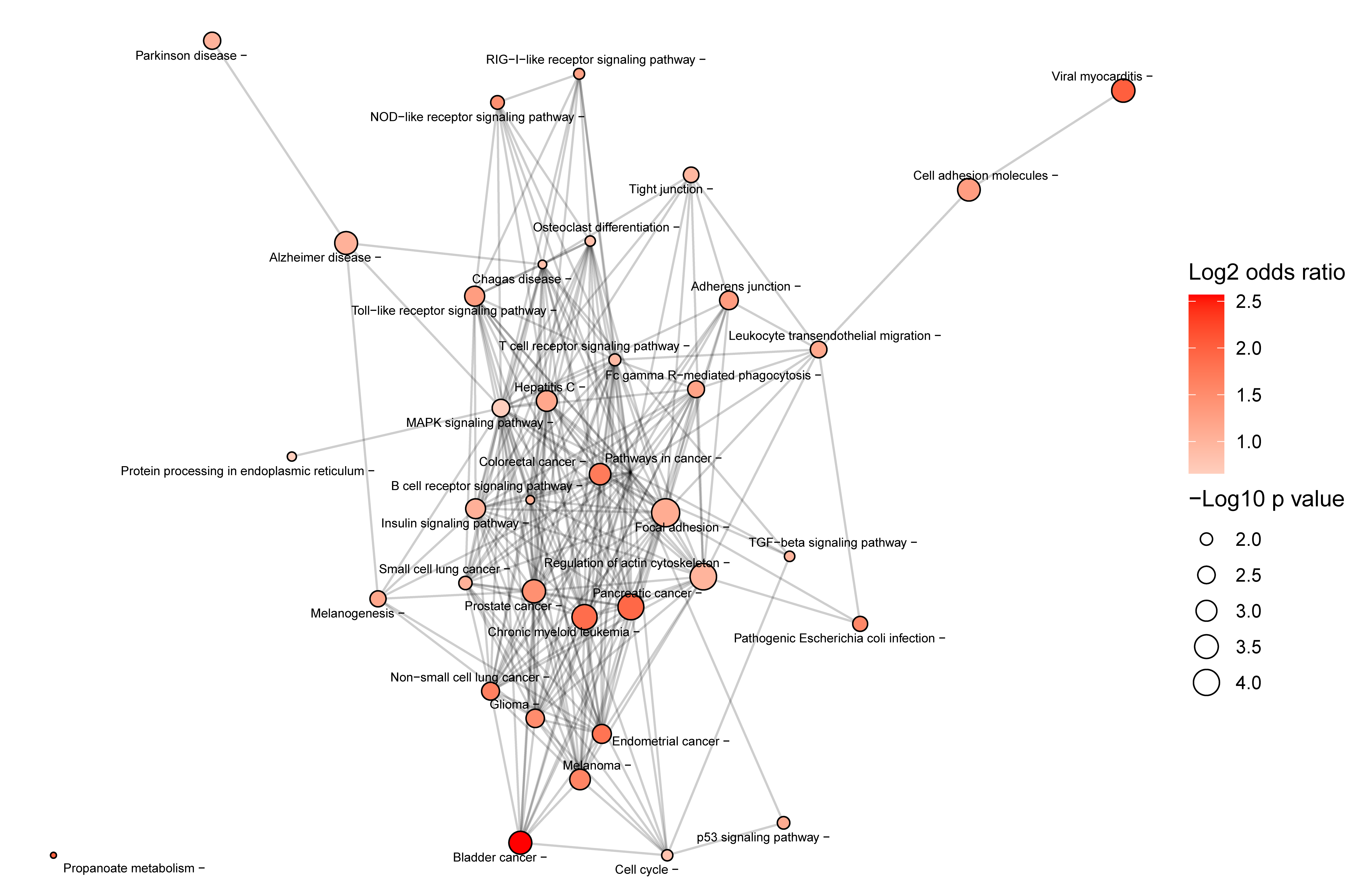

Supplement: Supplementary file 2 — Supplementary Information 2. [file 41598_2022_10866_MOESM2_ESM.tif]

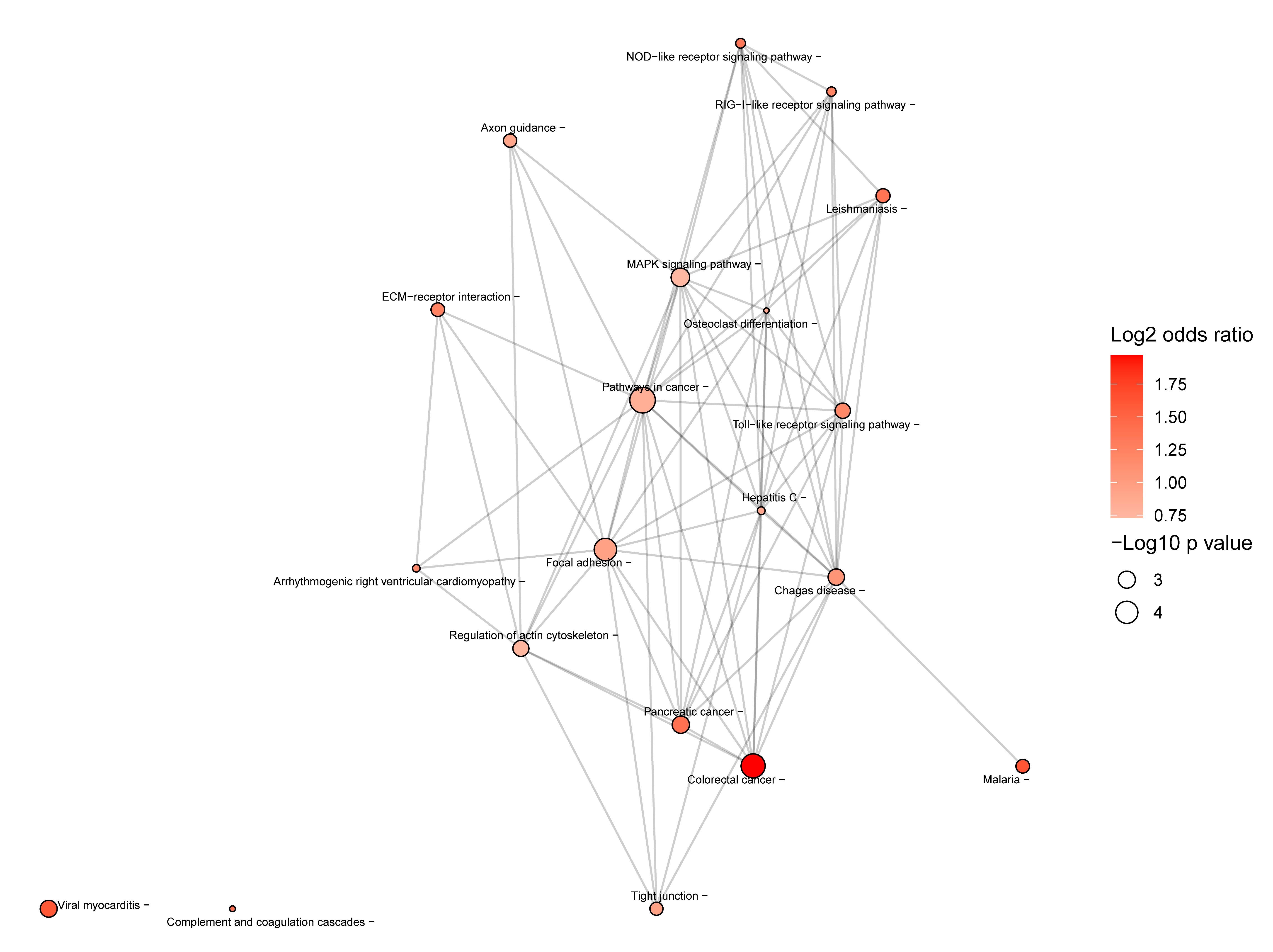

Supplement: Supplementary file 3 — Supplementary Information 3. [file 41598_2022_10866_MOESM3_ESM.tif]

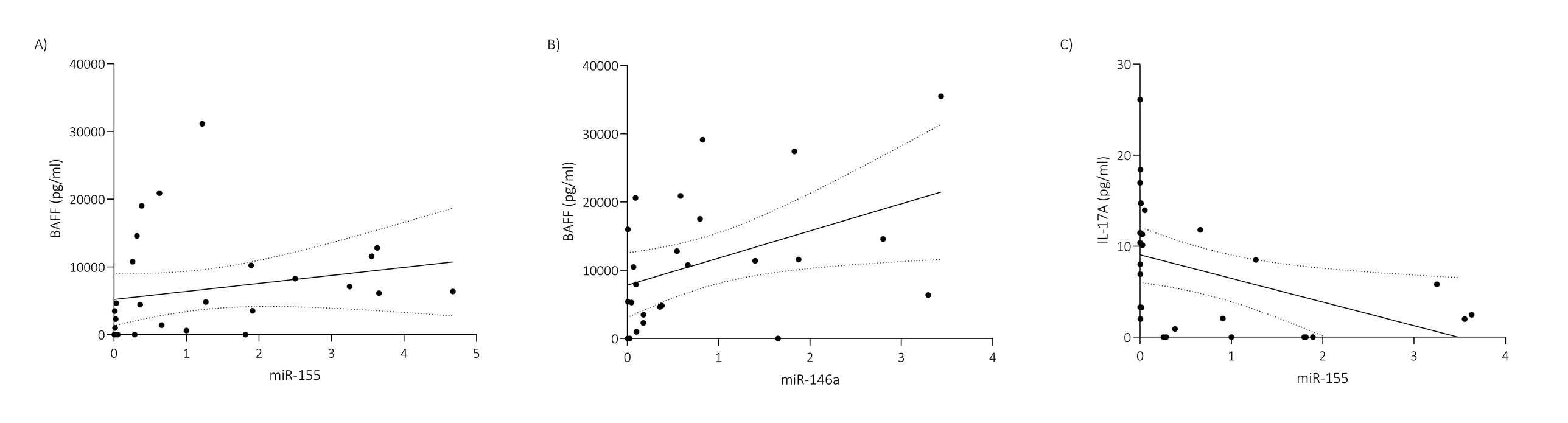

Supplement: Supplementary file 6 — Supplementary Information 6. [file 41598_2022_10866_MOESM6_ESM.tif]
