# Supplementary material for: Differential expression of circulating miRNAs after alemtuzumab induction therapy in lung transplantation
Source: Sci Rep. 2022 Apr 30;12:7072. doi: 10.1038/s41598-022-10866-w (PMC9056512; doi:10.1038/s41598-022-10866-w)
Supplement: Supplementary file 7 — Supplementary Information 7. [file 41598_2022_10866_MOESM7_ESM.docx]

Supplementary Tables

Table S1 – Serum levels of cytokines within the groups between the baseline and one after transplantation

| Timepoint |  | Groups | | | | | | |
| --- | --- | --- | --- | --- | --- | --- | --- | --- |
|  |  | Baseline | | | One year after transplantation | | | p-value |
|  |  | Median | Percentile 25 | Percentile 75 | Median | Percentile 25 | Percentile 75 |  |
| No-induction | TNF-α | 53.6 | 0.1 | 128 | 18.6 | 7.6 | 96.9 | 0.614 |
|  | **IL-13** | **39.1** | **30.7** | **47.7** | **0.1** | **0.1** | **27.8** | **0.002** |
|  | **IL-4** | **17.6** | **12.5** | **27.9** | **0.1** | **8.9** | **53.3** | **<0.001** |
|  | IL-10 | 6.9 | 1.7 | 28.6 | 3.6 | 0.1 | 20.1 | 0.184 |
|  | IL-6 | 38.6 | 14.2 | 88.3 | 70.5 | 13.2 | 125.2 | 0.460 |
|  | IL-2 | 37.9 | 0.1 | 149 | 94.7 | 65.6 | 245.8 | 0.132 |
|  | **IFN-γ** | **65.1** | **39.8** | **102.2** | **183** | **62.3** | **439.3** | **0.003** |
|  | IL-17A | 5.9 | 3.9 | 8.2 | 0.1 | 0.1 | 9.6 | 0.056 |
|  | IL-12p70 | 9.9 | 0.1 | 38.9 | 19.9 | 5.9 | 52.7 | 0.251 |
|  | APRIL | 29599 | 16331 | 42415 | 22855 | 14917 | 30712 | 0.074 |
|  | **BAFF** | **0.1** | **0.1** | **628** | **998** | **0.1** | **9586** | **0.008** |
|  | sCD40L | 50872 | 40818 | 69658 | 49173 | 33972 | 73564 | 0.788 |
|  | **IL-5** | **45.7** | **25.4** | **71.9** | **24.2** | **0.1** | **54.4** | **0.031** |
|  | **IL-9** | **21.8** | **16.7** | **29** | **9.1** | **0.1** | **23.2** | **0.006** |
|  | **IL-17F** | **26.8** | **12.3** | **36** | **0.1** | **0.1** | **20.5** | **0.001** |
|  | **IL-22** | **44.2** | **30.5** | **60.9** | **29.5** | **0.1** | **53.7** | **0.033** |
| Alemtuzumab | TNF-α | 54.7 | 11.6 | 239 | 21.4 | 2.1 | 88.9 | 0.255 |
|  | **IL-13** | **33.9** | **8.8** | **43.2** | **0.1** | **0.1** | **28.9** | **0.041** |
|  | **IL-4** | **11.5** | **0.1** | **20.1** | **0.1** | **0.1** | **0.1** | **0.005** |
|  | IL-10 | 6.4 | 1.8 | 14.1 | 5.3 | 0.1 | 11.5 | 0.406 |
|  | IL-6 | 47.4 | 14.2 | 86.4 | 34.6 | 17.6 | 76.5 | 0.683 |
|  | IL-2 | 58.5 | 0.1 | 191 | 132 | 0.1 | 314 | 0.360 |
|  | **IFN-γ** | **47.9** | **0.1** | **109.4** | **211.2** | **46.4** | **290.6** | **0.003** |
|  | **IL-17A** | **3.9** | **0.1** | **8.1** | **0.1** | **0.1** | **4.3** | **0.043** |
|  | IL-12p70 | 8.8 | 0.1 | 26.5 | 15.3 | 4.6 | 30.8 | 0.408 |
|  | APRIL | 26929 | 13387 | 56634 | 27696 | 16227 | 34670 | 0.668 |
|  | **BAFF** | **3934** | **0.1** | **9270** | **10232** | **4436** | **17518** | **0.015** |
|  | sCD40L | 65876 | 39363 | 87205 | 64790 | 93541 | 41697 | 0.877 |
|  | IL-5 | 28.1 | 0.1 | 40.2 | 10.8 | 0.1 | 55.5 | 0.275 |
|  | **IL-9** | **17.4** | **0.1** | **31.7** | **0.1** | **0.1** | **16.5** | **0.026** |
|  | IL-17F | 7.2 | 0.1 | 17.6 | 0.1 | 0.1 | 5.7 | 0.088 |
|  | **IL-22** | **22.8** | **3.9** | **38** | **0.1** | **0.1** | **16.5** | **0.015** |

Table S2 – Difference of serum levels of cytokines between the groups

| Timepoint |  | Groups | | | | | | |
| --- | --- | --- | --- | --- | --- | --- | --- | --- |
|  |  | No-induction | | | Alemtuzumab | | | p-value |
|  |  | Median | Percentile 25 | Percentile 75 | Median | Percentile 25 | Percentile 75 |  |
| Baseline | TNF-α | 53.6 | 0.1 | 128 | 54.7 | 11.6 | 239 | 0.392 |
|  | IL-13 | 39.1 | 30.7 | 47.7 | 33.9 | 8.8 | 43.2 | 0.113 |
|  | IL-4 | 17.6 | 12.5 | 27.9 | 11.5 | 0.1 | 20.1 | 0.111 |
|  | IL-10 | 6.9 | 1.7 | 28.6 | 6.4 | 1.8 | 14.1 | 0.818 |
|  | IL-6 | 38.6 | 14.2 | 88.3 | 47.4 | 14.2 | 86.4 | 0.176 |
|  | IL-2 | 37.9 | 0.1 | 149 | 58.5 | 0.1 | 191 | 0.822 |
|  | IFN-γ | 65.1 | 39.8 | 102.2 | 47.9 | 0.1 | 109.4 | 0.179 |
|  | IL-17A | 5.9 | 3.9 | 8.2 | 3.9 | 0.1 | 8.1 | 0.054 |
|  | IL-12p70 | 9.9 | 0.1 | 38.9 | 8.8 | 0.1 | 26.5 | 0.669 |
|  | APRIL | 29599 | 16331 | 42415 | 26929 | 13387 | 56634 | 0.589 |
|  | **BAFF** | **0.1** | **0.1** | **628** | **3934** | **0.1** | **9270** | **0.001** |
|  | sCD40L | 50872 | 40818 | 69658 | 65876 | 39363 | 87205 | 0.481 |
|  | **IL-5** | **45.7** | **25.4** | **71.9** | **28.1** | **0.1** | **40.2** | **0.019** |
|  | IL-9 | 21.8 | 16.7 | 29 | 17.4 | 0.1 | 31.7 | 0.232 |
|  | **IL-17F** | **26.8** | **12.3** | **36** | **7.2** | **0.1** | **17.6** | **0.002** |
|  | **IL-22** | **44.2** | **30.5** | **60.9** | **22.8** | **3.9** | **38** | **0.008** |
| One year after transplantation | TNF-α | 18.6 | 7.6 | 96.9 | 21.4 | 2.1 | 88.9 | 0.741 |
|  | IL-13 | 0.1 | 0.1 | 27.8 | 0.1 | 0.1 | 28.9 | 0.470 |
|  | IL-4 | 0.1 | 8.9 | 53.3 | 0.1 | 0.1 | 0.1 | 0.976 |
|  | IL-10 | 3.6 | 0.1 | 20.1 | 5.3 | 0.1 | 11.5 | 0.882 |
|  | IL-6 | 70.5 | 13.2 | 125.2 | 34.6 | 17.6 | 76.5 | 0.246 |
|  | IL-2 | 94.7 | 65.6 | 245.8 | 132 | 0.1 | 314 | 0.839 |
|  | IFN-γ | 183 | 62.3 | 439.3 | 211.2 | 46.4 | 290.6 | 0.742 |
|  | IL-17A | 0.1 | 0.1 | 9.6 | 0.1 | 0.1 | 4.3 | 0.298 |
|  | IL-12p70 | 19.9 | 5.9 | 52.7 | 15.3 | 4.6 | 30.8 | 0.393 |
|  | APRIL | 22855 | 14917 | 30712 | 27696 | 16227 | 34670 | 0.405 |
|  | **BAFF** | **998** | **0.1** | **9586** | **10232** | **4436** | **17518** | **0.011** |
|  | sCD40L | 49173 | 33972 | 73564 | 64790 | 93541 | 41697 | 0.301 |
|  | IL-5 | 24.2 | 0.1 | 54.4 | 10.8 | 0.1 | 55.5 | 0.402 |
|  | IL-9 | 9.1 | 0.1 | 23.2 | 0.1 | 0.1 | 16.5 | 0.352 |
|  | IL-17F | 0.1 | 0.1 | 20.5 | 0.1 | 0.1 | 5.7 | 0.296 |
|  | **IL-22** | **29.5** | **0.1** | **53.7** | **0.1** | **0.1** | **16.5** | **0.018** |

Table S3 - Serum levels of co-signaling immune molecules within the groups between the baseline and one after transplantation

| Timepoint |  | Groups | | | | | | |
| --- | --- | --- | --- | --- | --- | --- | --- | --- |
|  |  | Baseline | | | One year after transplantation | | | p-value |
|  |  | Median | Percentile 25 | Percentile 75 | Median | Percentile 25 | Percentile 75 |  |
| No-induction | sCD25 | 1287.8 | 833.4 | 2507.5 | 1206.6 | 906.5 | 1362.6 | 0.233 |
|  | 4-1BB | 59.5 | 28.3 | 77.0 | 61.6 | 39.5 | 98.0 | 0.391 |
|  | **sCD27** | **38161.1** | **29532.7** | **70727.4** | **91385.3** | **67772.1** | **139278.8** | **0.026** |
|  | **B7.2** | **66** | **58.9** | **95.9** | **152** | **114.9** | **192.1** | **0.001** |
|  | CTLA-4 | 3.4 | 0.6 | 7.1 | 6.6 | 1.2 | 9.2 | 0.233 |
|  | **PD-L1** | **37.9** | **30.7** | **43.7** | **48.2** | **36.6** | **55.2** | **0.042** |
|  | PD-L2 | 6079.7 | 4944.6 | 10050.6 | 6389.1 | 5543.2 | 9947.1 | 0.358 |
|  | PD-1 | 13.4 | 7.7 | 17.6 | 19.5 | 9.4 | 28.5 | 0.092 |
|  | Tim-3 | 29410.7 | 16164.2 | 46458.5 | 30771.8 | 13781.0 | 42898.8 | 0.845 |
|  | LAG-3 | 359.9 | 228.9 | 522.6 | 478.8 | 339.4 | 648.8 | 0.271 |
|  | Galectin-9 | 43828.4 | 32651.2 | 60192.4 | 40184.7 | 26008.6 | 73705.8 | 0.916 |
| Alemtuzumab | sCD25 | 1450.5 | 882.8 | 3114.7 | 1200.1 | 819.7 | 1640.7 | 0.264 |
|  | 4-1BB | 65.1 | 32.3 | 86.5 | 60.4 | 36.2 | 89.6 | 0.782 |
|  | sCD27 | 39674.5 | 10422.3 | 57245.3 | 57135.0 | 27393.3 | 94567.7 | 0.060 |
|  | B7.2 | 122.2 | 51.6 | 169.8 | 169.3 | 97.1 | 291.2 | 0.068 |
|  | CTLA-4 | 4.5 | 1.0 | 13.1 | 7.1 | 2.8 | 16.0 | 0.239 |
|  | PD-L1 | 42.5 | 31.2 | 56.7 | 53.7 | 31.9 | 69.2 | 0.431 |
|  | PD-L2 | 7753.1 | 4928.8 | 10346.9 | 8352.3 | 6434.0 | 10770.8 | 0.484 |
|  | PD-1 | 20.2 | 12.3 | 36.0 | 19.9 | 12.6 | 30.4 | 0.904 |
|  | Tim-3 | 31421.7 | 15828.5 | 40644.5 | 29773.3 | 15828.1 | 41838.2 | 0.829 |
|  | LAG-3 | 571.6 | 266.6 | 700.4 | 464.5 | 77.7 | 880.2 | 0.538 |
|  | Galectin-9 | 47058.3 | 32979.9 | 69466.7 | 49380.0 | 40645.7 | 72724.5 | 0.280 |

Table S4 – Summary of KEGG enriched pathways detected by enrichment analysis of the 4 significantly dysregulated miRNAs among the two groups at one year after transplantation

| **KEGG pathway name** | **KEGG identifier number** | **odds ratio** | **p-value** | **adjusted p-value (FDR: BH method)** |
| --- | --- | --- | --- | --- |
| Pathways in cancer | 05200 | 2.03 | 3.22E-07 | 7.31E-05 |
| Focal adhesion | 04510 | 2.15 | 3.44E-05 | 0.004 |
| Regulation of actin cytoskeleton | 04810 | 2.00 | 8.07E-05 | 0.006 |
| Pancreatic cancer | 05212 | 3.79 | 0.000 | 0.006 |
| Chronic myeloid leukemia | 05220 | 3.64 | 0.000 | 0.007 |
| Viral myocarditis | 05416 | 4.06 | 0.000 | 0.011 |
| Prostate cancer | 05215 | 2.74 | 0.000 | 0.011 |
| Bladder cancer | 05219 | 5.93 | 0.000 | 0.011 |
| Alzheimer disease | 05010 | 2.05 | 0.000 | 0.011 |
| Cell adhesion molecules | 04514 | 2.45 | 0.000 | 0.011 |
| Colorectal cancer | 05210 | 3.27 | 0.001 | 0.016 |
| Hepatitis C | 05160 | 2.25 | 0.001 | 0.019 |
| Melanoma | 05218 | 3.03 | 0.001 | 0.019 |
| Toll-like receptor sigling pathway | 04620 | 2.46 | 0.001 | 0.022 |
| Insulin sigling pathway | 04910 | 2.07 | 0.001 | 0.022 |
| Endometrial cancer | 05213 | 3.46 | 0.002 | 0.028 |
| Adherens junction | 04520 | 2.47 | 0.002 | 0.029 |
| Glioma | 05214 | 2.84 | 0.002 | 0.029 |
| Non-small cell lung cancer | 05223 | 3.14 | 0.003 | 0.033 |
| MAPK sigling pathway | 04010 | 1.58 | 0.003 | 0.034 |
| Parkinson disease | 05012 | 2.04 | 0.003 | 0.037 |
| Fc gamma R-mediated phagocytosis | 04666 | 2.33 | 0.004 | 0.038 |
| Leukocyte transendothelial migration | 04670 | 2.23 | 0.004 | 0.039 |
| Melanogenesis | 04916 | 2.26 | 0.005 | 0.043 |
| Tight junction | 04530 | 1.94 | 0.005 | 0.047 |
| Pathogenic Escherichia coli infection | 05130 | 2.97 | 0.006 | 0.051 |
| NOD-like receptor sigling pathway | 04621 | 2.74 | 0.008 | 0.066 |
| Small cell lung cancer | 05222 | 2.06 | 0.008 | 0.068 |
| p53 sigling pathway | 04115 | 2.16 | 0.010 | 0.075 |
| T cell receptor sigling pathway | 04660 | 1.89 | 0.010 | 0.079 |
| Cell cycle | 04110 | 1.76 | 0.012 | 0.084 |
| RIG-I-like receptor sigling pathway | 04622 | 2.37 | 0.012 | 0.084 |
| Maturity onset diabetes of the young | 04950 | 0.20 | 0.013 | 0.084 |
| TGF-beta sigling pathway | 04350 | 2.00 | 0.013 | 0.084 |
| Osteoclast differentiation | 04380 | 1.81 | 0.013 | 0.084 |
| Protein processing in endoplasmic reticulum | 04141 | 1.58 | 0.015 | 0.090 |
| B cell receptor sigling pathway | 04662 | 2.10 | 0.015 | 0.090 |
| Chagas disease | 05142 | 1.81 | 0.015 | 0.090 |
| Propanoate metabolism | 00640 | 3.95 | 0.017 | 0.096 |

Table S5 – Summary of KEGG enriched pathways detected by enrichment analysis of the 3 significantly dysregulated miRNAs in alemtuzumab group one year after transplantation compared to baseline

| **KEGG pathway name** | **KEGG identifier number** | **odds ratio** | **p-value** | **adjusted p-value (FDR: BH method)** |
| --- | --- | --- | --- | --- |
| Pathways in cancer | 05200 | 1.76 | 1.42E-05 | 0.003 |
| Colorectal cancer | 05210 | 3.92 | 3.51E-05 | 0.004 |
| Focal adhesion | 04510 | 1.94 | 8.56E-05 | 0.006 |
| MAPK sigling pathway | 04010 | 1.66 | 0.001 | 0.031 |
| Pancreatic cancer | 05212 | 2.63 | 0.001 | 0.039 |
| Viral myocarditis | 05416 | 3.03 | 0.001 | 0.039 |
| Chagas disease | 05142 | 2.10 | 0.001 | 0.040 |
| Regulation of actin cytoskeleton | 04810 | 1.67 | 0.001 | 0.040 |
| Toll-like receptor sigling pathway | 04620 | 2.29 | 0.002 | 0.045 |
| Leishmaniasis | 05140 | 2.59 | 0.003 | 0.054 |
| ECM-receptor interaction | 04512 | 2.36 | 0.003 | 0.054 |
| Malaria | 05144 | 3.07 | 0.003 | 0.054 |
| Axon guidance | 04360 | 1.89 | 0.003 | 0.054 |
| Tight junction | 04530 | 1.93 | 0.003 | 0.054 |
| NOD-like receptor sigling pathway | 04621 | 2.53 | 0.006 | 0.095 |
| RIG-I-like receptor sigling pathway | 04622 | 2.33 | 0.007 | 0.095 |
| Hepatitis C | 05160 | 1.84 | 0.008 | 0.097 |
| Arrhythmogenic right ventricular cardiomyopathy | 05412 | 2.28 | 0.008 | 0.097 |
| Complement and coagulation cascades | 04610 | 2.64 | 0.009 | 0.097 |
| Osteoclast differentiation | 04380 | 1.81 | 0.009 | 0.097 |

Table S6 – NormFinder analysis

| **Rank** | **Gene name** | **Stability value** |
| --- | --- | --- |
| 1 | RNU6 | 0.056 |
| 2 | hsa-miR-24 | 0.066 |
| 3 | hsa-miR-30b | 0.068 |
| 4 | hsa-miR-17 | 0.072 |
| 5 | hsa-miR-125a | 0.074 |
| 6 | hsa-miR-486 | 0.086 |
| 7 | hsa-miR-21 | 0.123 |
| 8 | hsa-miR-146a | 0.148 |
| 9 | hsa-miR-23b | 0.198 |
| 10 | hsa-miR-98 | 0.199 |
| 11 | hsa-miR-155 | 0.203 |
| 12 | hsa-miR-182 | 0.219 |
| 13 | hsa-miR-99a | 0.247 |
| 14 | hsa-miR-31 | 0.271 |
